# PD01 Advances In The Incorporation Of Medical Devices: A Case Study On Technology Developed By A Brazilian University

**DOI:** 10.1017/S0266462325101396

**Published:** 2025-12-29

**Authors:** Fotini Toscas, Nadja Mayrink, Vania Cristina Canuto Santos, Luciene Bonan, Eduardo Dias

## Abstract

**Introduction:**

The National Committee for Health Technology Incorporation (CONITEC) plays a crucial role in advising the Brazilian Ministry of Health. Evaluating medical devices (MDs) poses challenges due to gaps in clinical and economic evidence. This study aimed to: (i) describe the current landscape, highlighting advancements in specific health technology assessment (HTA) processes for medical devices; and (ii) deepen knowledge through a case study.

**Methods:**

The study methodology included descriptive exploratory research, reviewing normative references, and conducting documentary analysis on the evaluation of technologies for incorporating medical devices into Brazil. The normative review involved consulting the CONITEC website and “Sistema Saúde Legis,” which compiles federal regulations. Document analysis used CONITEC’s “CONITEC in Numbers” panel filtered by “health products,” which tracks the progress, incorporation status, and updates on health technologies over the last 12 years. The case study selection criteria included demand with a decision on incorporation, external claimant profile, and suitability for reassessment, which allowed comparative analysis of the HTA processes.

**Results:**

Since its creation, CONITEC has received 122 requests, approximately 10 percent of which are related to MDs. Of the total evaluated, 65 percent came from external applicants and 35 percent from areas of the Ministry of Health. The study highlighted significant changes in the structure and configuration of CONITEC in 2022, including the establishment of subcommittees and thematic committees specifically dealing with MDs. A case study on dynamic phototherapy, developed at a Brazilian University, illustrates the evolution: initially not recommended in 2020, dynamic phototherapy received a favorable recommendation in 2023 following review. This underscores the importance of specialist involvement and real-world data collection.

**Conclusions:**

CONITEC’s 12-year journey has improved MD evaluation, especially with the new specialized committees. The study demonstrates the evolution of the HTA process in Brazil, emphasizing the importance of specialized subcommittee participation and contextual data collection for comprehensive evaluation of demands.

